# How do lipid-based drug delivery systems affect the pharmacokinetic and tissue distribution of amiodarone? A comparative study of liposomes, solid lipid nanoparticles, and nanoemulsions

**DOI:** 10.22038/IJBMS.2024.75152.16292

**Published:** 2024

**Authors:** Farnaz Khaleseh, Mohammad Barzegar-Jalali, Parvin Zakeri-Milani, Zahra Karami, Mohammad Reza Saghatchi Zanjani, Hadi Valizadeh

**Affiliations:** 1 Student Research Committee, Faculty of Pharmacy, Tabriz University of Medical Sciences, Tabriz, Iran; 2 Department of Pharmaceutics, Faculty of Pharmacy, Tabriz University of Medical Sciences, Tabriz, Iran; 3 Pharmaceutical Sciences Research Center, Health Institute and School of Pharmacy, Kermanshah University of Medical Sciences, Kermanshah, Iran; 4 Drug Applied Research Center and Faculty of Pharmacy, Tabriz University of Medical Sciences, Tabriz, Iran; 5 Liver and Gastrointestinal Diseases Research Center and Faculty of Pharmacy, Tabriz University of Medical Sciences, Tabriz, Iran; 6 Department of Pharmaceutical Nanotechnology, School of Pharmacy, Zanjan University of Medical Sciences, Zanjan, Iran; 7 Pharmaceutical Nanotechnology Research Center, Zanjan University of Medical Sciences, Zanjan, Iran

**Keywords:** Amiodarone, Liposome, Nanoemulsion, Pharmacokinetic parameter, Solid lipid nanoparticle

## Abstract

**Objective(s):**

Lipid-based drug delivery systems (DDS) can improve the pharmacokinetic (PK) parameters of some drugs. Especially those with a high volume of distribution (Vd) leading to off-target accumulation and toxicity. Amiodarone as an anti-arrhythmic agent induces hypothyroidism and liver disorders limiting its clinical indication.

**Materials and Methods:**

In the present study, amiodarone PK parameters and biodistribution after IV administration of four nano-formulations to rats were compared. The formulations were liposomes, solid lipid nanoparticles (SLN), PEGylated SLN (PEG-SLN), and nanoemulsions (NE). All formulations were optimized.

**Results:**

The nanoparticles were spherical with a diameter of 100-200 nm and sustained *in vitro* drug release in buffer pH 7.4. The best-fitted model for the plasma concentration-time profile was two-compartmental. *In vivo* studies indicated the most changes in PKs induced after liposome, SLN, and NE administration, respectively. The area under the curve (AUC) and maximum plasma concentration (C_max_) of liposomes, SLN, and NE were 22.5, 2.6, 2.46 times, and 916, 58, and 26 times higher than that of amiodarone solution, respectively (*P-value*<0.05). The heart-to-liver ratio of amiodarone was higher for nano-formulations compared to drug solution except for liposomes.

**Conclusion:**

Lipid-based particles can improve the PK parameters of amiodarone and its distribution in different tissues.

## Introduction

Over the past few decades, different drug delivery systems (DDS) and nanomedicines have attracted researchers’ attention in clinical and non-clinical fields. The carrier’s effect on pharmacokinetic (PK) parameters of the encapsulated drug for example results in higher blood concentration, longer circulation time, higher diffusion to target site, improvement of drug efficacy, and reduced side effects. These changes lead to improvement in drug efficacy. The effects on PK parameters depend on the physicochemical properties of the drug delivery system. Indeed, the composition and formulation of the drug delivery system determine the alteration of PK parameters (1). One group of DDS is lipid-based nanoparticles. Liposomes, solid lipid nanoparticles (SLN), and nanostructured lipid carriers (NLC) are the most studied lipid-based nanoparticles. They have high stability, high capacity for drug loading, and can be prepared at large scale (2). The different component structure of lipid-based nanoparticles and drug solubility indicates that a wide range of drug molecules can be loaded in different lipid-based nanoparticles. Liposomes are one of the most interesting nanoparticles composed of phospholipids and cholesterol (Chol). Chol existence in the membrane affects the packing density of phospholipids and fluidizes the liposome membrane. Therefore, Chol is necessary for the preparation of stable and controlled-release liposomes (3). Both hydrophilic and lipophilic drugs can be loaded in liposomes. They can improve drug permeability through membranes. They decrease the chemical and biological degradation of loaded drugs (4). Drug encapsulation into liposomes reduces drug distribution to different tissues. Therefore, a lower volume of distribution (V_d_) leads to lower adverse effects (5). Encapsulation into liposomes reduces drug clearance (Cl) and increases the half-life (t_1/2_) of the drug in most cases. Liposomes are suitable carriers for targeting, and the particle size can be controlled according to the purpose and target tissue. They can be functionalized by different targeting ligands that specifically deliver the drug to target tissues. Researchers demonstrated *in vitro* and *in vivo* studies of long-acting targeted liposomes of paclitaxel. The results showed selective distribution to the target tissue, drug accumulation in tumor tissue, prolongation of drug residence time in blood, and improvement of cellular uptake. Therefore, paclitaxel liposomal formulation exhibited improved efficacy, reduced toxicity, and side effects (6). Other members of lipid-based nanoparticles are SLNs. The components of this colloidal system are biocompatible lipids. The lipid phase is solid at body temperature. The emulsifier molecules in the SLN formulation stabilize the structure. SLNs can control drug release, especially for low water-soluble drugs (7). The stable structure of SLNs overcomes drug leakage occurring through liposome membranes. SLNs have a high capacity for drug loading. The loading capacity depends on drug solubility in the lipid phase. They also protect loaded drugs from degradation (8). SLNs can affect the drug pharmacokinetics (PKs). As the study demonstrated, erlotinib-loaded SLNs increased the drug bioavailability about 2 folds in comparison to free Erlotinib (9). The process of PEGylation in solid lipid nanoparticles (SLNs) prevents their detection by the mononuclear phagocyte system (MPS), resulting in a longer circulation time for the loaded drug. This modification was shown to enhance drug effectiveness, as demonstrated by Koide *et al*. In their research, PEGylated lipid nanoparticles showed a significantly higher survival rate compared to non-PEGylated lipid nanoparticles when administered to toxin-treated mice (10). Nanoemulsion (NE) can be defined as the dispersion of nano-size droplets in a continuous phase. NE can be prepared by dispersing a liquid lipid in water resulting in oil in water NE. This structure has some similarities to SLNs. The physical state of the lipid and its composition determine the *in vitro* and *in vivo* characteristics of the formulation. The encapsulated molecule and lipid structure should be considered for the selection of the optimum carrier. For example, quercetin showed a higher loading percentage and bioaccessibility in NE and NLC compared to SLNs (11). But curcuminoids exhibited higher area under the curve (AUC) and maximum plasma concentration (C_max_) after administration of SLNs compared to NLC; although entrapment efficiency (EE) of NLC was higher (12).

Low water-soluble drugs, belonging to class II of BCS, can be good candidates for encapsulation into lipid-based DDS. In this category, amiodarone as an anti-arrhythmic agent has a limited dissolution rate and bioavailability due to low water solubility (13). Amiodarone is administered for the management of different arrhythmias like atrial fibrillation. It has a long circulation time and high V_d_. Therefore, the drug accumulates in off-target tissues (such as the liver, lung, and thyroid) leading to the manifestation of adverse effects (14). As shown in previous studies, improvement of *in vitro* drug release, *in vivo* PKs, and efficacy of amiodarone was achieved through encapsulation into different carriers like polymeric nanoparticles and lipid-based nanoparticles (15-17).

Despite the good therapeutic effects of amiodarone, low water solubility, inappropriate PKs like high V_d_, side effects, and toxicities limit the clinical indication of amiodarone. These parameters can be improved by encapsulation into suitable carriers. This study aimed to assess how lipid-based DDS affect the properties of amiodarone both *in vitro* and *in vivo*. Specifically, the impact of liposomes, SLN, PEG-SLN, and NE on the PK parameters of amiodarone was investigated. To achieve this, amiodarone was loaded into the lipid-based DDS mentioned above. The formulations were optimized based on *in vitro* tests and their physicochemical properties were evaluated. The optimum formulations, as determined through these evaluations, were administered to rats via intravenous (IV) injection. The PK parameters of amiodarone in all formulations were evaluated. Also, the tissue distribution of amiodarone in each formulation was assessed between the liver, heart, kidney, and spleen. A tissue distribution study was conducted to understand how the carrier changed the drug distribution throughout the body.

## Materials and Methods

***Materials***

Soy lecithin (SL), glyceryl monostearate (GMS), cholesterol (Chol), Witepsol W 35, Miglyol 812 N, Poloxamer 188, Tween 80, SLS, Myrj 52, ZnSO4, formic acid, and amiodarone hydrochloride were procured from Sigma Aldrich Co (MO, USA). Chloroform, HPLC grade acetonitrile, and methanol were purchased from Merck (Darmstadt, Germany). All materials were used intact and without further purification.

***Animals***

Twenty male Sprague Dawley rats (200±16 g) were provided by the Pasteur Institute, Iran branch (Karaj, Iran). The rats were placed in separated cages at 25±2 ^°^C and 60±5% humidity under 12 hr light/dark cycles during one week before the experiment. The animal research was conducted based on the principles of the Guide for the Care and Use of Laboratory Animals. The study protocol received approval from the Ethics Committee of Tabriz University of Medical Sciences (Tabriz, Iran) with a project number of IR.TBZMED.VCR.REC.1397.259.

***Preparation of nano-formulations***

**Figure 1:**
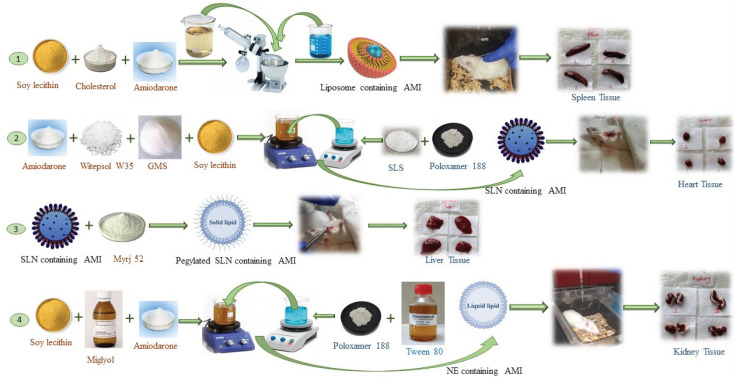
Preparation procedure of 1) liposomal amiodarone, 2) amiodarone loaded SLNs, 3) amiodarone loaded PEG-SLNs, 4) amiodarone loaded NE

The preparation procedure of nano-formulations of amiodarone is depicted in Figure 1. 

*Preparation of liposomes*

The liposomal formulation was prepared by thin film hydration as elucidated in previous studies with some modifications (18, 19). SL, Chol, and amiodarone in the ratios mentioned in Table 1 were dissolved in chloroform to obtain a transparent solution. The solution was heated to evaporate the organic solvent at 60 ^°^C under vacuum using a rotary evaporator (Heidolph Co., Germany). Following the solvent evaporation, a film was obtained. Nitrogen flow was applied to remove the organic solvent residues. The film was exposed to distilled water for 1 hr to be hydrated, and liposomes were prepared. During the hydration, the mixture was further homogenized using a water bath sonicator for 40 min, followed by 10 min of probe sonication (Bandelin, Germany). Formulation 6 with the smallest particle size was selected to be extruded through a 100 nm membrane at 60 ^°^C. The prepared liposomes were stored at 4 °C until further analysis.

*Preparation of SLNs and PEG-SLNs*

The amiodarone-loaded SLNs were prepared by the hot homogenization method reported previously with some modifications (20, 21). The lipid phase composed of 230 mg of Witepsol W 35, 25 mg of GMS, and 17.5 mg of SL was melted at 70 ^°^C. Amiodarone (50 mg) was dissolved in the melted lipid phase. Poloxamer (5 mg) and SLS (2 mg) were dissolved in 10 ml water and then heated up to 70 ^°^C. The aqueous phase was added to melted lipid by shaking. The prepared emulsion was homogenized by high shear homogenization (Heidolph, Silent Crusher M, Germany) at 15000 rpm for 10 min. The emulsion was sonicated for 4 min by probe sonication (Bandelin, Germany) to reduce the droplet size. The nano-size droplets of lipids were solidified under 1 hr of stirring at room temperature. PEG-SLNs were prepared using the same procedure, but polyoxyethylene stearate (Myrj 52) was added to the lipid phase. Final formulations were stored at 4 ^°^C until further analysis.

*Preparation of NE*

The emulsification was accomplished according to previous reports with some modifications (22). Poloxamer 188 and labrasol/Tween 80 were dissolved in water at 70 ^°^C according to Table 2. The solution was added to the oil phase containing amiodarone, SL, and Miglyol at the same temperature. The mixture was homogenized using a high shear homogenizer (Heidolph, Silent Crusher M, Germany) at 21000 rpm for 10 min. The emulsion was sonicated by probe sonication (Bandelin, Germany), and droplet size was reduced during 10 min. 

***In vitro evaluation of nano-formulations***

*Particle size, PDI, zeta potential, and morphology study*

The particle size and polydispersity index (PDI) of the formulations were evaluated using dynamic light scattering (Malvern Instruments, Malvern, UK), applying a 90-plus particle sizer at 25 ^°^C. The scattering angle was fixed at 90 ^°^. The surface charge of particles (zeta potential) was measured by the same zeta sizer instrument.

The morphology of particles was observed using Leo 906 transmission electron microscopy (TEM), Carl Zeiss AG, 100 kv (Oberkochen, Germany). The samples were diluted with deionized water (1:20) followed by staining with 2% uranyl acetate. A drop of the samples was dried on a carbon-coated copper grid and observed (23).

*Entrapment efficiency (EE)*

EE was evaluated indirectly for liposomes, SLNs, and PEG-SLNs. The un-entrapped drug of liposomes was separated by the dialysis method at 4 ^°^C. A distinct amount of liposomal formulation was poured into a dialysis cassette and floated in distilled water. After 10 hr, the free drug was diffused through the membrane and detected by high-performance liquid chromatography (HPLC) according to the method explained below. 

The free drug of SLNs and PEG-SLNs was separated using amicon (Ultra-4, Millipore, USA). Therefore, the top of the membrane was filled with SLN suspension followed by centrifugation at 5000 rpm for 8 min. The solution containing un-loaded amiodarone was analyzed by HPLC. 

EE was evaluated using the following equations:

EE% = 100 * (Initial drug used – Free drug) / Initial drug used

*In vitro drug release study*

*In vitro* release study of amiodarone from all formulations was assessed by performing a dialysis cassette (cut off 10 kDa) in one compartment rotating cell. The donor part contained liposome, SLN, PEG-SLN, or NE formulations equivalent to 5 mg amiodarone. Each cassette was submerged in 120 ml phosphate buffer saline (pH=7.4, 37 ^°^C) containing 0.8% Tween 80 as stabilizer, and stirred at 70 rpm. At defined time intervals (30, 60, 90, 120, 240, 360, 420, 1080, and 1440 min) 1 ml of the medium was replaced by fresh medium to mimic the sink condition. The withdrawn sample was analyzed by HPLC to plot the cumulative drug release profile versus time. 

The release kinetic of samples was assessed by mathematical models: zero-order, first-order, Hixson Crowell, Korsmeyer-peppas, and Higuchi. The decision was based on linear regression. The model with the highest regression coefficient (R^2^ value) was selected as the best-fitted model (24).

***Quantitative determination of amiodarone by HPLC***

Knauer liquid chromatographic system (Germany) equipped with ultraviolet spectrophotometric detector was applied and set at 242 nm. The analytical column was an RP-chromolith speed rod end-capped 50×4.6 mm, and the analysis was conducted at room temperature. The system EZChrom Elite software was applied for data achievement, analysis, and reporting. The mobile phase was composed of 60% acetonitrile and 40% distilled water adjusted to pH 4 by formic acid. The isocratic flow rate was set at 2 ml/min. The ICH guidelines for bioanalytical validation of the method were conducted for validation of the method according to system suitability, linearity, precision, accuracy, limits of detection (LOD), and quantification (LOQ). RSD % <15% was accepted for plasma samples. All analysis was repeated three times (25).

*Preparation of standard and working solution*

The primary stock solution was prepared by dissolving 25 mg of amiodarone in 25 ml of methanol with a final concentration of 1000 µg/ml. It was kept at 4 ^°^C in the dark. This solution was stable for at least three months in the mentioned condition. Every day, the stock solution was diluted with methanol to prepare working solutions. These solutions were then spiked into rat plasma with a final volume of 100 µl. The final concentration range for presentation of the calibration curve was 50-500 ng/ml.

***In vivo studies in rats***

*PKs study*

In the experiment conducted on Sprague Dawley rats, the PKs of amiodarone nanoparticles were assessed. The rats were divided into five groups at random (4 rats in each group). Group I received a liposomal formulation (equivalent to 12.5 mg/kg of amiodarone). Groups II, III, and IV received the equivalent of 6.25 mg/kg of amiodarone of SLN, PEG-SLN, and NE, respectively. The amiodarone solution was injected into group V as the control (6.25 mg/kg). The formulations were prepared freshly, and they were filtered through 0.22 µm syringe filters before IV administration for sterilization. 

The experimental protocol was based on the literature with some modifications (26, 27). The jugular vein of each rat was cannulated one day before drug administration. For this purpose, each rat was anesthetized by administering a mixture of ketamine and xylazine via intraperitoneal (IP) injection (at a dosage of 100:10 mg/kg). The right jugular vein of the rats was cannulated using a polyethylene cannula with a wall thickness of 0.008 inches, an inner diameter of 0.023 inches, and an outer diameter of 0.038 inches. The cannula was then connected to a sampling head made of silicone rubber tubing, which had a wall thickness of 0.011 inches, an inner diameter of 0.025 inches, and an outer diameter of 0.047 inches. On the following day, the amiodarone formulations were intravenously injected through the catheter connected to the jugular vein.

The blood samples (600 µl) were collected at specific time intervals (15, 30, 60, 120, 360, and 720 min) following administration of formulations through a catheter attached to the jugular vein. To separate the plasma, the samples were subjected to centrifugation at 1500 g for 10 min, after which the plasma was stored at -20 ^°^C until analysis. Amiodarone concentration in plasma samples was evaluated by HPLC and the plasma concentration-time profile was plotted. The PK parameters were calculated according to the plasma concentration-time profile and the related data were analyzed using Thermo Scientific Kinetica software (version 5.0). 

*Preparation of plasma samples*

Methods including liquid-liquid extraction and protein precipitation (solvent precipitation by acetonitrile and methanol, solvent-salt precipitation, and acid precipitation) were performed for preparation of plasma samples to find the method with the highest recovery % (28-30). To calculate recovery %, the ratio of the peak area of extracted amiodarone from plasma samples to the peak area of un-extracted amiodarone in the mobile phase at the same concentration was evaluated. As presented in Table 3, the highest recovery % was obtained by the solvent-salt precipitation method. In this method, 100 µl of plasma was mixed with zinc sulfate: acetonitrile and vortexed 2 min. Then centrifuged at 12000 rpm for 12 min, and the transparent liquid was injected into HPLC.

*Biodistribution studies*

The rats were sacrificed 12 hr post-injection of formulations, for evaluation of amiodarone distribution in different tissues. The liver, spleen, kidney, and heart were collected from each rat. The organs were washed with saline, and the excess fluid of tissues was removed with paper towels and weighed. The organs were stored at -20 ^°^C until analysis by HPLC. For drug assay, each tissue was homogenized and suspended in equal weight of saline. After preparation of the solution of each tissue, sample proteins were precipitated by zinc sulfate (1.16 M): acetonitrile: sample (1:4:5), and the drug concentration was analyzed by HPLC (31). 

*PK modeling*

The plasma concentration-time profile for each group was plotted to find the best PK model. The groups contained rats receiving liposome, SLN, PEG-SLN, NE, and amiodarone solution. The data of the plasma concentration-time profile were analyzed using Thermo Kinetica (version 5.0). Noncompartmental, one, two, and three-compartmental open models were evaluated to find the best-fitted model. The visual inspection, the Aikaike Information Criterion (AIC), and residuals (mean differences of observed and calculated values for each model) were applied to find the fit adequacy of the model. 

***Statistical analysis***

The obtained data were analyzed by analysis of variance (ANOVA) with *post-hoc* Tukey and Kruskal Wallis test. They were based on normal distribution of data and homogeneity of variances. Therefore, differences in PK parameters between various treatment and control groups were evaluated. The level of significance was considered as 0.95 confidence interval. The data were reported as mean±SEM or mean±SD.

## Results

***Preparation of nano-formulations***

The amiodarone-loaded liposomes were prepared by the thin film hydration method. Smaller particle sizes and higher drug concentrations were the criteria of the optimum formulation. As presented in Table 1, formula number 6 was the optimum formulation. The volumetric diameter of liposomes was 450 nm and 101.1 nm, before and after extrusion, respectively. The SLNs and PEG-SLNs were fabricated according to the hot homogenization method and Myrj 52 was added to the lipid phase for preparing PEG-SLNs. Table 2 shows the ratio of formulations for preparing amiodarone NE. The optimum formulation based on smaller droplet size was formula number 6.

***In vitro evaluation of nano-formulations***

*Particle size, PDI, zeta potential, morphology studies, and EE%*

The mean diameter, PDI, and zeta potential of optimum formulations have been elucidated in Table 4. The sizes of all formulations were in the range of 100-200 nm. The smallest particle size was obtained for liposomal formulation (101.1 nm). The largest particle size was obtained for PEG-SLN (199.7 nm). The size distribution of nanoparticles is as important as particle size; because particle size affects drug distribution in the body. The PDI elucidated the size distribution of particles. The studied nanoparticles showed narrow size distribution indicating homogeneity of formulations. 

The nano-formulations had a negative surface charge except SLN. It elucidated 40.2 mV zeta potential. The pH of Poloxamer and SlS solution was 7-9.5. On the other hand, the estimated pKa of GMS was 13. When pKa>pH, the charge is positive. The result of the present study was in accordance with previous studies and the surface charge of SLNs containing Poloxamer 188, GMS, and lecithin was positive. Also, the glycerol group in the lecithin structure could induce a positive charge to Witepsol nanoparticles (32, 33).

**Figure 2:**
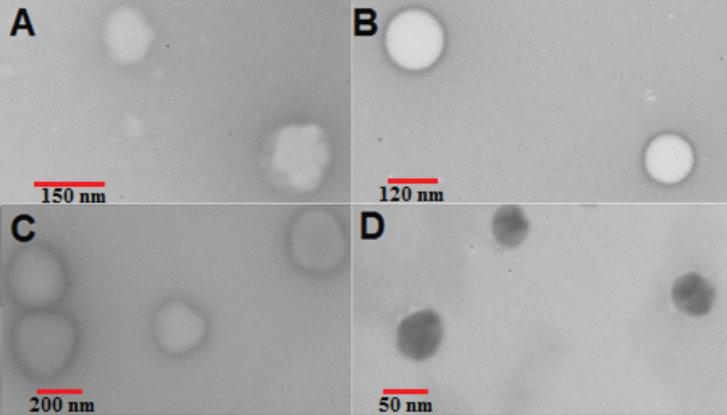
Morphology of nano-formulations of amiodarone by TEM. A) Liposome. B) SLN. C) PEG-SLN. D) NE

TEM: Transmission electron microscopy; PEG-SLN: PEGylated solid lipid nanoparticles; SLN: Solid lipid nanoparticles; NE: Nanoemulsion

The morphology of liposomes, SLNs, PEG-SLNs, and NE was observed by TEM images as presented in Figure 2. The particles were spherical and no aggregation was observed. The size of particles was in accordance with DLS results.

The SLNs have a higher loading capacity for amiodarone as a low water-soluble drug. SLNs and PEG-SLNs had an EE% value of 99%, while EE% for liposomes was 83.5%.

*In vitro drug release profile of nano-formulations*

**Figure 3:**
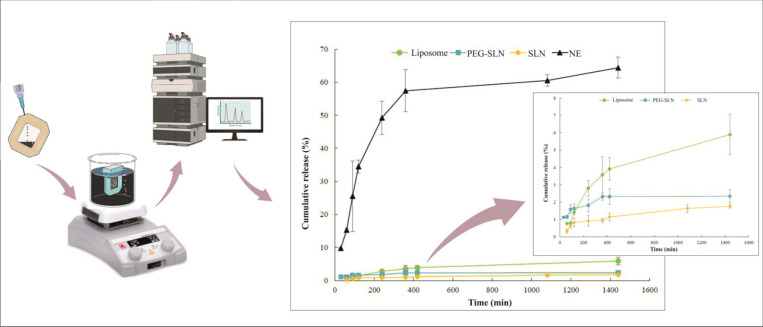
Amiodarone release profile of liposomes, SLNs, PEG-SLNs, and NE at phosphate buffer pH 7.4 and 37 ^°^C (n = 3)

PEG-SLN: PEGylated solid lipid nanoparticles; SLN: Solid lipid nanoparticles; NE: Nanoemulsion

The* in vitro* release profile of amiodarone from liposome, SLNs, PEG-SLNs, and NE over 24 hr, at pH=7.4 and 37 ^°^C is depicted in Figure 3. After 24 hr, the cumulative release of amiodarone from liposomes, SLNs, PEG-SLNs, and NE reached 5.89%, 1.64%, 2.34%, and 60.54%, respectively. A biphasic drug release pattern was obtained for NE formulation, with burst release during the first 4 hr followed by sustained release. The solubility difference of amiodarone in the lipid phase of liposome, SLN, and NE could be the reason for the variable release rate from formulations. 

The kinetics of release patterns were evaluated for formulations. The selection was based on the higher R^2 ^value indicated in Table 5. The best-fitted model for liposomes and SLNs was Higuchi, meaning that cumulative drug release was proportional to the square root of time. This model shows that the initial amiodarone concentration in the matrix of particles is higher than its solubility. Also, it elucidates that drug diffusion took place in one dimension and constant rate. (34-36). The Korsmeyer Peppas model elucidated the mechanism of drug release in PEG-SLN and NE. In the Peppas model, the fraction of drug released at time t is equal to K*t^n^. K and n show the release rate constant and the release exponent, respectively. The value of n defines the release mechanisms. The n value for PEG-SLN was 0.22 indicating the Fickian diffusion mechanism and 0.74 for NE, indicating non-Fickian transport of amiodarone from nanoparticles (37). 

***Method validation for amiodarone detection in plasma samples***

A simple, fast, and reliable method for evaluating the amiodarone plasma levels was validated. The method was validated according to system suitability, linearity, accuracy, precision, LOD, and LOQ. The detail information of method validation is presented in Table 6. The method was linear in the concentration range of 50-500 ng/ml with acceptable inter and intra-day accuracy and precision. The LOD and LOQ values of the method were 11.96 and 36.23 ng/ml, respectively. The retention time of amiodarone was 2.1 min with no interference with other peaks. The method was fast, which helped to perform PK studies in a short period of time. The LOD and LOQ of the HPLC method were lower than previous studies and it was applicable to a wide range of concentrations (38).

***PKs study***

**Figure 4:**
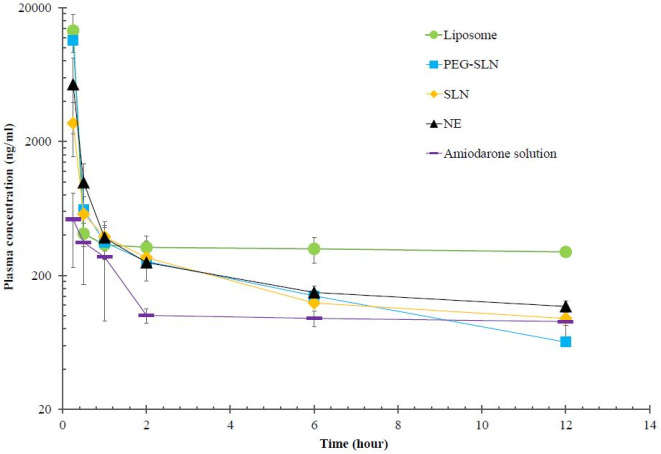
Mean plasma concentration-time data (log-linear scale) after IV administration of liposomes, SLNs, PEG-SLNs, NE, and free drug to rats (n=4)

The plasma drug concentration-time profile for all formulations was investigated by non-compartmental and compartmental analysis. Figure 4 shows the mean plasma concentration-time data after IV administration of liposome, SLN, PEG-SLN, NE, and free drug to rats. The fast declining slope after administration shows that the compartmental model of PKs fits better to the data. Different compartmental model analysis was performed based on the AIC and residuals, and mean values are presented in Table 7. Fitting the data to the three-compartmental model was not suitable. The best fit for IV administration of formulations was reached with a two-compartmental model according to the smaller AIC and residuals. 

Both AUC and C_max_ were normalized based on 6.25 mg/kg of amiodarone in this study. As presented in Table 8, nanoparticles of amiodarone reached higher drug exposure AUC compared to amiodarone solution. The AUC of liposomal formulation was higher compared to other formulations (*P*-value<0.05). The AUC values after administration of all formulations were 22.5, 4.6, 2.6, and 2.5 times higher for liposomal formulation, PEG-SLN, SLN, and NE compared to amiodarone solution, respectively. The value for the NE and SLN group was higher compared to the drug solution significantly but the difference between PEG-SLN and drug solution was not significant (*P*-value = 0.51). Compared to SLN, PEG-SLN showed higher AUC, C_max_, Cl, and V_d_. Among SLN, PEG-SLN, and NE, lower AUC and C_max_ were reached by NE. On the other hand, higher plasma concentrations may result in more drug accessibility to reach target sites and improved drug efficacy. The normalized C_max _in groups receiving liposomal amiodarone, PEG-SLN, SLN, and NE was significantly higher compared to the amiodarone solution, respectively. Also, the comparison of nanoparticle groups showed significantly higher C_max _for liposomal amiodarone in comparison with other nanoparticles. The results showed that the drug Cl was reduced 19.6, 2.8, 2, and 1.3 times after administration of liposomes, SLNs, NE, and PEG-SLNs compared to the drug solution, respectively. The differences in Cl were not significant statistically in different formulations. The t_1/2_ of liposomal amiodarone was significantly higher than that of other formulations. Surprisingly the t_1/2_ was significantly lower for SLN and PEG-SLN compared to amiodarone solution. The V_d_ of the drug was reduced after administration of nano-formulations in comparison to the drug solution. Although the differences were not significant, lower V_d_ may result in lower adverse effects and improved efficacy. The lower amiodarone distribution indicated that more drug was accessible to reach the target site as it had not been distributed to off-target organs.

***Biodistribution studies***

**Figure 5:**
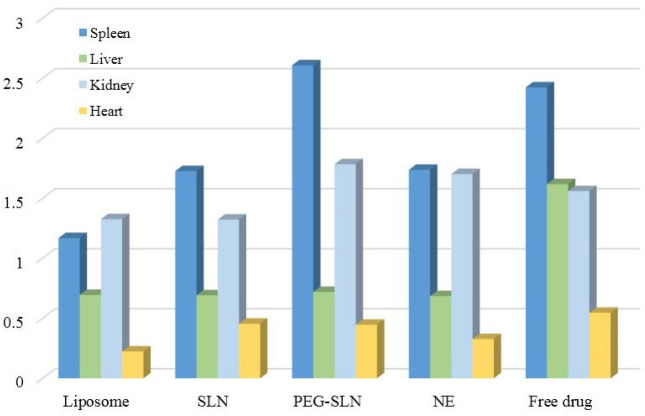
Tissue distribution of amiodarone after liposome, SLN, PEG-SLN, NE, and amiodarone solution injection according to the amount of drug per tissue weight

PEG-SLN: PEGylated solid lipid nanoparticles; SLN: Solid lipid nanoparticles; NE: Nanoemulsion

The drug concentration in the spleen, liver, kidney, and heart was measured 12 hr after IV injection of the formulations to assess tissue distribution (Figure 5). The drug was distributed statistically equally to the spleen, liver, heart, and kidney after IV administration of the drug solution. However, the liposomal formulation accumulated more in the kidneys than in the spleen, liver, and heart. After administration of SLN and NE formulations, amiodarone was distributed to the spleen significantly more than to the other tissues. As the target tissue of amiodarone was the heart, the heart-to-liver ratio of drug concentration could be a comparative index. Heart to liver ratio of amiodarone for drug solution was 0.34 while it was 0.32, 0.66, 0.62, and 0.48 for liposomes, SLNs, PEG-SLNs, and NE, respectively. It showed that the drug accumulated in the target tissue compared to the liver after administration of SLNs, PEG-SLNs, and NE.

**Table 1:** Ratio of components for formulation optimization of amiodarone loaded liposome

Number	SL:Chol ratio	Lipid:drug ratio	Drug conc. (mg/ml)	Size (nm)
1	4:1	15:1	0.6	No homogenous film
2	5:1	12:1	0.3	1100
3	5:1	9:1	1.25	740
4	4:1	6:1	0.5	No homogenous film
5	4:1	18:1	0.2	933
6	3:1	12:1	0.8	450
7	2.5:1	14:1	0.4	791

PEG-SLN: PEGylated solid lipid nanoparticles; SLN: Solid lipid nanoparticles; NE: Nanoemulsion

**Table 2:** Ratio of components for formulation preparation of amiodarone nanoemulsion (NE)

Formulation number	Amiodarone (mg)	SL (W/V %)	Poloxamer (W/V %)	Labrasol (W/V %)	Tween 80 (W/V %)	Miglyol (V/V %)	Size (nm)
1	20	1.3 %	0.85 %	0.85 %	-	10 %	839
2	20	1.1 %	0.78 %	1.1 %	-	10 %	592
3	20	1.6 %	1.2 %	3.2 %	-	20 %	720
4	20	2.6 %	1.7 %	-	1.7 %	20 %	460
5	-	2.6 %	1.7 %	-	1.7 %	20 %	158
6	10	2.6 %	1.7 %	-	1.7 %	20 %	163

SL: Soy lecithin

**Table 3:** Different preparation methods of plasma sample for HPLC analysis

Method	Material	Material:plasma	Recovery %
Liquid extraction	Chloroform	1:1	17.9 %
Acid precipitation	Perchloric acid (15 % V/V):NaOH (3 M)	3:2:10	No detection
Acid precipitation	Perchloric acid 15 % V/V	1:2	No detection
Solvent precipitation	Acetonitrile	3:1	63.5 %
Solvent-salt precipitation	Zinc sulfate (1.16 M): Acetonitrile	1:4:5	90 %
Solvent-salt precipitation	Zinc sulfate (1.16 M): Acetonitrile	1:4:10	51.9 %

**Table 4:** Particle size, PDI, surface charge, and EE% of optimum formulation of amiodarone liposome, SLN, PEG-SLN, and NE

Nano-formulation	Size (nm)	PDI	Zeta (mV)	EE %
Liposome	101.1	0.36	-46.9	83.5%
SLN	117.5	0.16	40.2	99.1%
PEG-SLN	199.7	0.23	-13.9	99.2%
NE	163.9	0.14	-38.9	-

PDI: Poly dispersity index; EE: Entrapment efficiency; SLN: Solid lipid nanoparticles; PEG-SLN: PEGylated solid lipid nanoparticles; NE: Nanoemulsion

**Table 5:** Release kinetic of amiodarone (R2 value) at 37 ^°^C phosphate buffer pH 7.4

Formulation	Zero-order	First order	Higuchi	Korsmeyer-Peppas	Hixson Crowell
Liposome	0.8009	0.8074	0.9301	0.9176	0.8053
SLN	0.8874	0.8888	0.9493	0.8842	0.8883
PEG-SLN	0.5072	0.5077	0.6858	0.8425	0.5075
NE	0.6261	0.709	0.7799	0.8478	0.6815

**Table 6:** Details of HPLC method validation. The method was sensitive and accurate enough for nano-range concentrations of amiodarone in rat plasma samples

Validation parameter	Definition	Detail of results				
Linearity	The least square regression method was conducted for calculation of calibration curve linearity. The calibration curves were plotted in the range of 50–5000 ng/ml 3 times.	Intercept	Slope		Correlation	
		242.42	18.11		0.999	
Precision Accuracy	Precision is the deviation degree of the acquired responses after multiple evaluations reported by percentage of relative standard deviation (% RSD). Accuracy is the deviation of the acquired concentration from the actual concentration.	Intra-day	Added conc. (ng/ml)	Precision (RSD %)		Accuracy (%)
			50	0.05		97.52
			250	6.76		84.82
			500	14.16		90.74
		Inter-day	50	5.17		94.11
			250	7.75		94.03
			500	1.58		100.95
System suitability	Factor	Obtained value		Recommended value		
	Plate count (N)= 16 × (retention time/peak width)^2^	1020		Higher		
	Capacity factor (K´) = (retention time of analyte - retention time of un-retained compound/retention time of un-retained compound)	0.6		0.5 < K´ < 10		
	Tailing factor (T)= W_0.05 _⁄ 2f	1.12		0.5 ≤ T ≤ 2		
Limit of detection (LOD)	3.3 × (standard deviation of y-intercept/slope of regression line)	11.96 ng/ml				
Limit of quantitation (LOQ)	10 × (standard deviation of y-intercept/slope of regression line)	36.23 ng/ml				

**Table 7:** Aikaike information criterion (AIC) and residual values for selection of the best-fitted model for pharmacokinetic (PK) analysis

Group	Mean value of residuals for 1 compart. model	Mean value of residuals for 2 compart. model	Mean value of AIC for 1 compart. model	Mean value of AIC for 2 compart. model
Liposome	0.938	0.003	1233.213	-40.986
SLN	0.558	0.041	28.987	-1.968
PEG-SLN	0.979	0.039	96.783	-5.348
NE	1.033	0.060	86.250	-6.859
Free drug	0.192	0.049	14.913	-3.861

PEG-SLN: PEGylated solid lipid nanoparticles; SLN: Solid lipid nanoparticles; NE: Nanoemulsion

**Table 8:** Pharmacokinetic (PK) parameters of amiodarone nano-formulations after IV administration of 12.5 mg/kg of liposomal amiodarone and 6.25 mg/kg SLN, PEG-SLN, NE of amiodarone, and drug solution to rats (n = 4). The calculated values were based on the two-compartmental model as the best-fitted model

Group	Liposome	SLN	PEG-SLN	NE	Free drug
PK parameter					
AUC (mg*min/L)^a^	4447.03±1395.88	516.81±48.13	901.96±691.13	486.46±163.03	197.71±34.37
k_el_ (1/hr)	0.010±0.001	0.113±0.019	0.134±0.033	0.084±0.019	0.042±0.005
t_½_ (hr)	72.36±10.05	6.47±1.03	6.05±1.83	9.49±2.72	16.81±7.00
Cl (ml/min/kg)	1.66±0.40	12.33±1.27	24.90±14.21	15.87±4.71	32.56±5.71
Vz (L/kg)	9.73±1.63	6.91±1.21	17.44±12.94	13.17±4.69	48.33±13.77
C_max_ ( g/ml)^a^	1154.39±439.81	73.79±23.73	171.79±158.08	33.08±22.14	1.26±0.47

^a^ Dose-normalized (based on 6.25 mg/kg of amiodarone) values were compared for statistical analysis.

PEG-SLN: PEGylated solid lipid nanoparticles; SLN: Solid lipid nanoparticles; NE: Nanoemulsion; AUC: Area under the curve; Cl: Clearance; Cmax, Maximum plasma concentration; t_1/2_, Half-life

## Discussion

The lipid-based DDS have gained researchers’ attention in recent era. They changed both *the in vitro* release profile of the encapsulated drug and *in vivo* characteristics including PK parameters, drug distribution, and efficacy. As there are different physicochemical characteristics for each kind of these DDS, they can change PK parameters differently. Amiodarone is an effective medication for atrial fibrillation, the most common cardiac arrhythmia. The clinical usage of amiodarone has been limited because of its off-target toxicity. By reducing of V_d_ of drugs, the efficacy may improve and side effects could be limited. The present study aimed to evaluate the carrier effect on PK parameters of amiodarone. For this purpose, liposome, SLN, PEG-SLN, and NE formulations of amiodarone were optimized and *in vitro* characteristics were assessed. *In vitro* study revealed that particle size of formulations was in the range of 100–200 nm, and particles had narrow size distribution. Previous studies showed that 10 nm gold particles were distributed in different organs like the thymus, testis, kidney, lung, spleen, heart, liver, and brain in rats compared to 50 and 250 nm nanoparticles that were only distributed in the spleen and liver. Therefore, polydisperse particles had variable distributions in the body. The smaller values of PDI indicate a narrow size distribution as presented for studied nanoparticles in this study while values more than 0.5 show a broad size distribution that is not desirable (39). 

The surface charge and hydrophobicity of particles affect both *in vitro* and *in vivo* behavior of nanoparticles. Nanoparticles are exposed to proteins and small molecules in biological environments and adsorb them to reduce their surface energy. The function of nanoparticles is limited by opsonization. The physicochemical characteristics of nanoparticles effect on the formation of this corona leads to different interactions between nanoparticles and cells and tissues. The most pronounced influence was induced by the surface charge. Negative charge led to a higher number of bound proteins compared to positively charged nanoparticles (40). Electrostatic interactions are the main driving forces for protein adsorption onto different particles in most cases (41). Although hydrophobic interactions are responsible for these interactions as proved in the study conducted by Rezwan *et al*., it was found that the same charged proteins and particles were adsorbed despite the electrostatic repulsion (42). Other studies showed that proteins with low isoelectric points preferably are adsorbed onto positively charged particles (43). As reported, the positive charge led to higher drug uptake because of stronger electrostatic attraction or improved particle enterocyte interactions. The interaction between the particle and the negative charge of the cell surface led to higher absorption from GI. Hydrophobicity influences particle recognition by the immune system in the body. PEGylation hinders the particles from macrophage attack; therefore, the residence time of the drug in the body increases. The surface charge has an important role in formulation stability. The particles with no surface charge tend to aggregate during storage; while positive or negative particles repel each other resulting in stable formulations (44, 45). According to reported data, the surface charge of SLNs composed of Witepsol W 35 depends on the surfactants. The existence of the glycerol group in the chemical structure of lecithin in the present study could induce a positive charge for SLNs. Incorporation of PEG molecules on the surface of nanoparticles reduced the charge due to higher hydrophilicity (32, 46). 

The mechanism of drug release from NE was non-Fickian transport as it was elucidated by the Korsmeyer Peppas model. Amiodarone was released from NE formulation during two phases. First, burst release of amiodarone occurred up to 4 hr and it was followed by the sustained release phase. The burst release showed that the drug was accumulated at the interface of droplets. The solubility of amiodarone in the lipid phase of NE was another important factor effective in drug release. The low capacity of Miglyol for dissolving amiodarone resulted in equal amiodarone tendency to the water phase and lipid phase. The release medium was adjusted at pH=7.4 to mimic the physiological condition; although amiodarone indicated the least release at this pH according to the literature (17). As shown in Figure 3, amiodarone was released from SLNs very slowly. The reason could be the high solubility of amiodarone in lipids of SLN leading to a higher tendency of the drug to particles compared to the release medium. Another study indicated that drug release was sustained in both SLN and NLC formulations, although liquid lipids resulted in more drug release than solid lipids, as shown in the present study (47). Dolatabadi *et al*. stated that both SLN and NLC induced prolonged *in vitro* release of curcuminoids. The formulations improved PK parameters including AUC and C_max_ compared to free curcuminoids after administration to mice (12). The presence of PEG molecules on the surface of particles induced weaker interactions between amiodarone and lipid matrix. It resulted in higher release rates of drugs from PEG-SLN compared to SLN (48-50). The leaky membrane of liposome induced more drug release rate compared to SLNs. Sustained drug release could be a reason for higher plasma concentrations of amiodarone after administration of nanoformulations. As presented in Table 8, NE showed the lowest C_max_, while amiodarone was released from NE at a faster rate compared to other nanoparticles. It means that SLNs and PEG-SLNs induced more sustained drug release that led to higher plasma concentrations and drug exposure during 12 hr. Higher plasma concentrations result in more drug accessibility at target sites, which improves drug efficacy. The* in vivo* assessments of the present study in rats indicated that liposomal amiodarone resulted in the highest AUC and C_max_ and lowest Cl compared to other nanoformulations. The higher AUC values are probable when the drug stays in solubilized form in circulation for longer periods. As amiodarone metabolism was through hepatic CYP2C8 and CYP3A3/4, the lower liver distribution could lead to higher plasma concentrations of amiodarone. As observed, amiodarone distribution to the liver was limited by the preparation of nano-formulations. Administration of amiodarone nanoformulations resulted in lower V_d_ and lower liver uptake and increased C_max _compared to amiodarone solution. The highest C_max_ was observed for liposomes, then PEG-SLNs, SLNs, and NE (Table 8). The results presented that clearance of the drug was reduced after administration of nanoparticles; although the differences were not significant. Drug release from nanoparticles could affect the clearance of drugs from the body. Due to sustained drug release from nanoparticles, clearance of amiodarone was lower after administration of nanoparticles compared to amiodarone solution. The t_1/2 _of liposomal amiodarone was significantly higher than that of all other formulations, while surprisingly it was significantly lower for SLN, PEG-SLN, and NE compared to the control group. The reason could be high plasma concentrations of amiodarone after administration of nanoparticles. By the way, more studies are required to understand the exact reason. 

A previous study proved that liposomal amiodarone (3 mg/kg) in rats improved drug efficacy and resulted in shorter duration of lethal arrhythmias and mortality (51). Another study showed that liposomal formulation increased AUC and residence time of amiodarone in blood circulation about 5 and 8 times. Also, liposomes reached specifically cardiomyocytes when cardiac radiofrequency ablation was applied (52). The results of another study indicated that improvement of amiodarone solubility by preparation of self nano emulsifying drug delivery system improved drug bioavailability. The low water solubility of amiodarone limited its *in vivo* efficacy. By preparing self nano emulsifying drug delivery system, amiodarone AUC and C_max_ increased, while Cl and V_d_ decreased (53). Researchers evaluated the effect of particle charge on PKs and the biodistribution of drugs. The negative particles showed better bioavailability than positive charge particles with no difference in tissue distribution (54). Another study reported that positive charge nanoparticles less than negative particles were accumulated in the liver leading to higher t_1/2 _of drug (55). In the present study differences in amiodarone distribution to liver were not significant among groups receiving positive SLNs and negative PEG-SLNs (Figure 5). In the study conducted by Tiwari and Pathak, PK parameters of drug-loaded NLC and SLNs were compared. It was found that NLC had higher EE compared to SLN due to the space provided by liquid lipids in the particle structure. The liquid lipid provided crystal defects in the nanoparticle structure, and drug molecules could be entrapped into these imperfections. The* in vivo* study confirmed 4.9 and 2.7-fold higher AUC in groups receiving NLC in comparison to simvastatin suspension and SLN, respectively. NLC resulted in lower V_d_ compared to SLN formulation, and the V_d_ of both groups was lower than the group receiving non-encapsulated drugs. Smaller V_d_ means that a larger fraction of the drug remained in the central compartment to reach the target site of action (56). 

In this study, SLNs, liposomes, PEG-SLNs, and NE had lower V_d_ compared to the amiodarone solution. As amiodarone shows high t_1/2_ and V_d_, off-target toxicity could appear. By preparing nano-formulations, the reduction of V_d_ results in more drug efficacy as it is accessible to the heart as a high-perfusion organ. Besides, t_1/2_ decreased for SLN and PEG-SLN but increased for liposomes compared to amiodarone solution. Also, the assessment of previous studies showed that the effectiveness of a carrier on PK parameters depends on the encapsulated drug and the components used for preparation of the particles. The PK parameters of amiodarone changed the most for liposomes in the lipid-based particles in this study. 

## Conclusion

Amiodarone was formulated into four lipid-based nanoparticles including liposomes, SLNs, PEG-SLNs, and NE. The composition of each formulation was optimized to obtain a smaller particle size. The* in vitro* drug release study indicated that SLNs, PEG-SLNs, liposomes, and NE sustained drug release from nanoparticles. The* in vivo* PKs study confirmed that the best-fitted model for formulations followed the two-compartmental model. It was found that nano-formulations of amiodarone improved PK parameters compared to amiodarone solution including higher AUC and C_max_. Among SLN, PEG-SLN, and NE, the highest C_max_ was reached by PEG-SLN. Amiodarone release from NE was higher than other formulations. The distribution study showed that the heart-to-liver ratio for SLNs, PEG-SLNs, and NE was higher than that of the amiodarone solution. Also, drug accumulation in the spleen for free drug and PEG-SLN was higher than for liposomes, SLNs, and NE. Amiodarone distribution to kidneys after liposome and SLN administration was lower than other formulations. The results showed that liposomes resulted in more changes in PK parameters than SLNs, PEG-SLNs, and NE as lipid-based nanoparticles. 

## Authors’ Contributions

F K, H V, M BJ, and P ZM designed the experiments; F K, Z K, and MR SZ performed experiments and collected data; F K, H V, and M BJ discussed the results and strategy; F K prepared the manuscript; H V and P ZM edited the article; H V, M BJ, and P ZM supervised, directed, and managed the study; F K, H V, M BJ, P ZM, Z K, and M R SZ approved the final version to be published.

## Conflicts of Interest

The authors declare no conflicts of interest or personal relationships that could influence this research.
